# Supplementary material for: Atmospheric-river-induced foehn events drain glaciers on Novaya Zemlya
Source: Nat Commun. 2024 Aug 15;15:7021. doi: 10.1038/s41467-024-51404-8 (PMC11327257; doi:10.1038/s41467-024-51404-8)
Supplement: Supplementary file 1 — Supplementary Information [file 41467_2024_51404_MOESM1_ESM.pdf]

# Supplementary information to:

# Atmospheric-river-induced foehn events drain glaciers on Novaya Zemlya

*J. Haacker, B. Wouters, X. Fettweis, I.A. Glissenaar, J.E. Box*

Tab. S1: Overview and comparison of glacier mass change trends over different periods derived with different methods.

| Period | I/O model | GRACE/GRACE-FO | ICESat | CryoSat-2 |
|---|---|---|---|---|
| | | ⟵ all values in $\mathrm{Gt\,yr^{-1}}$ ⟶ | | |
| 1980–2022 | $-5.7 \pm 2.9$ | | | |
| 1981–2010 | $-5.3 \pm 2.7$ | | | |
| 2002–2022 | $-7.1 \pm 3.2$ | $-8.7 \pm 0.6$ | | |
| 2003–2009 | $-4.4 \pm 3.0$ | $-8.7 \pm 1.4$ | $-7.8 \pm 3.0$ | |
| 2011–2022 | $-12.1 \pm 3.6$ | $-12.5 \pm 0.7$ | | $-12.5 \pm 1.6$ |

Tab. S2: Glacier surface elevation change rates for different focus-regions in Novaya Zemlya. The table compares the in Moholdt *et al.* [1, para. 13] earlier reported values to those of this study.

| Region | Area | 2003–2009 | 2011–2022 |
|---|---|---|---|
| ice divide (5 km) | $8218\,\mathrm{km^2}$ | $0.09\,\mathrm{m\,yr^{-1}}$ | $-0.27\,\mathrm{m\,yr^{-1}}$ |
| lower elevations | $7483\,\mathrm{km^2}$ | $-0.92\,\mathrm{m\,yr^{-1}}$ | $-1.16\,\mathrm{m\,yr^{-1}}$ |
| marine fronts | $4482\,\mathrm{km^2}$ | $-0.94\,\mathrm{m\,yr^{-1}}$ | $-1.32\,\mathrm{m\,yr^{-1}}$ |
| land fronts | $3001\,\mathrm{km^2}$ | $-0.89\,\mathrm{m\,yr^{-1}}$ | $-0.91\,\mathrm{m\,yr^{-1}}$ |
| Barents side | $10\,931\,\mathrm{km^2}$ | $-0.46\,\mathrm{m\,yr^{-1}}$ | $-0.77\,\mathrm{m\,yr^{-1}}$ |
| Kara side | $10\,391\,\mathrm{km^2}$ | $-0.25\,\mathrm{m\,yr^{-1}}$ | $-0.49\,\mathrm{m\,yr^{-1}}$ |

Tab. S3: List of dates in 1990–2022 with more than 1 Gt surface melt water production according to MAR. For each day we indicate whether we find foehn winds based on transects of daily average air temperature, wind speeds, and humidity from CARRA. Further, we indicate the wind direction by "E", "W", or "other" for easterlies, westerlies, or other wind directions, respectively (see Methods in main article).

| Date | Melt [$\mathrm{Gt\,d^{-1}}$] | Direction | Foehn |
|---|---|---|---|
| 1996-07-22 | 1.0 | W | yes |
| 1996-07-27 | 1.2 | E | yes |
| 1996-07-28 | 1.2 | E | yes |
| 1996-07-29 | 1.1 | E | yes |
| 1997-07-08 | 1.1 | E | yes |
| 1998-07-15 | 1.1 | E | yes |
| 2000-07-15 | 1.1 | W | yes |
| 2000-08-13 | 1.1 | E | yes |
| 2000-08-14 | 1.3 | E | yes |
| 2002-07-07 | 1.1 | W | no |
| 2002-08-17 | 1.1 | E | yes |
| 2003-06-23 | 1.0 | E | yes |
| 2003-08-18 | 1.2 | E | yes |
| 2004-07-04 | 1.2 | W | yes |
| 2004-07-05 | 1.4 | W | yes |
| 2004-07-06 | 1.1 | W | yes |
| 2004-07-09 | 1.1 | W | yes |
| 2004-07-18 | 1.0 | E | yes |
| 2004-08-02 | 1.3 | W | yes |
| 2005-08-27 | 1.1 | W | yes |
| 2006-07-26 | 1.0 | other | no |
| 2007-06-30 | 1.0 | E | yes |
| 2013-07-20 | 1.3 | E | yes |
| 2013-08-08 | 1.2 | W | no |
| 2015-07-30 | 1.2 | E | yes |
| 2015-07-31 | 1.2 | E | yes |
| 2015-08-01 | 1.1 | E | yes |
| 2016-07-04 | 1.4 | W | no |
| 2016-07-05 | 1.1 | W | no |
| 2016-07-13 | 1.1 | E | yes |
| 2016-07-14 | 1.3 | E | yes |
| 2016-07-15 | 1.4 | E | yes |
| 2016-07-19 | 1.2 | other | no |
| 2016-07-21 | 1.2 | W | yes |
| 2016-08-20 | 1.4 | W | yes |
| 2016-08-21 | 1.3 | other | no |
| 2016-09-11 | 1.0 | E | yes |
| 2017-07-20 | 1.1 | other | no |
| 2018-07-20 | 1.1 | W | yes |
| 2018-07-21 | 1.2 | W | yes |
| 2018-07-24 | 1.1 | W | yes |
| 2019-08-20 | 1.0 | E | no |
| 2020-07-03 | 1.1 | E | yes |
| 2020-07-04 | 1.1 | E | yes |
| 2020-07-05 | 1.0 | E | yes |
| 2020-07-06 | 1.1 | E | yes |
| 2020-07-07 | 1.1 | E | yes |
| 2020-07-31 | 1.0 | E | yes |
| 2020-08-01 | 1.6 | E | yes |
| 2020-08-02 | 1.1 | E | yes |
| 2020-08-03 | 1.2 | E | yes |
| 2020-08-16 | 1.0 | E | yes |
| 2021-08-03 | 1.2 | E | yes |
| 2021-08-05 | 1.0 | E | yes |

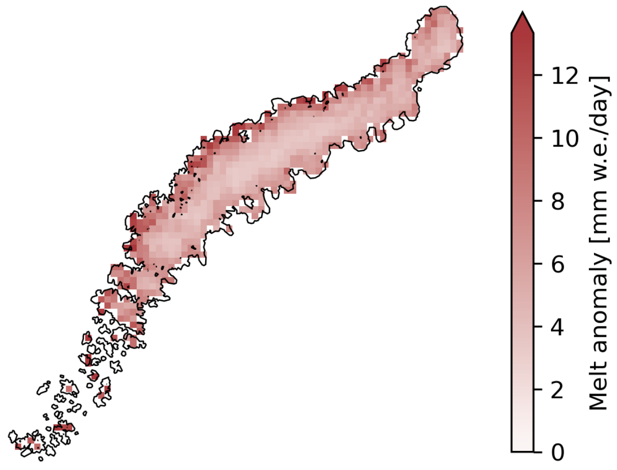


Fig. S1: Average MAR daily surface melt water production anomaly 2011–2022 during melt-season.

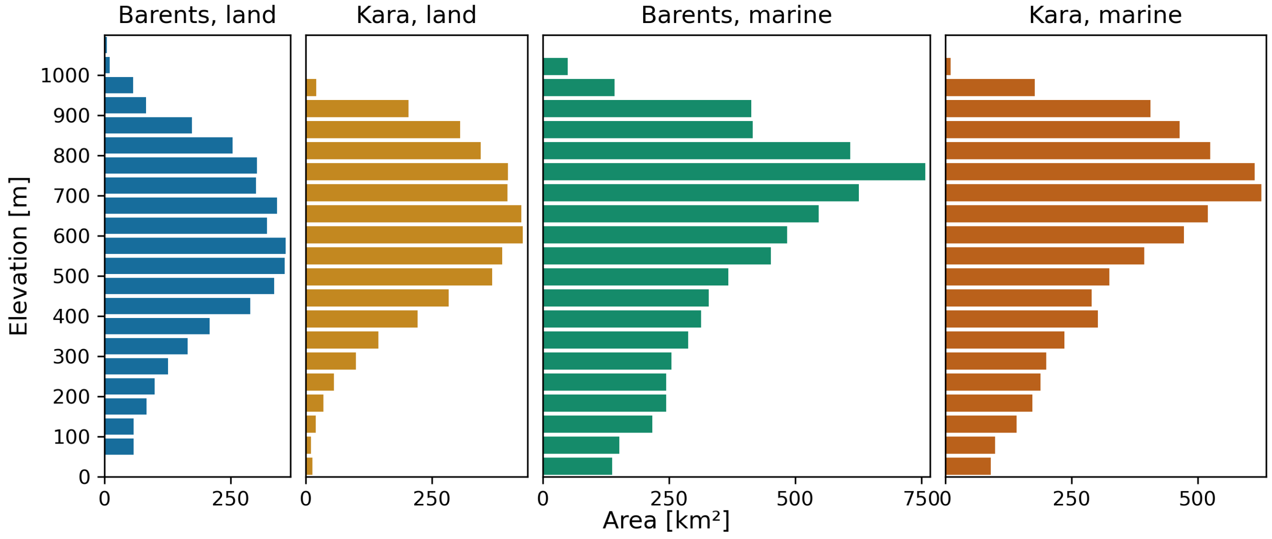


Fig. S2: Hypsometry of glacierized area for categories of termination-type and coast.

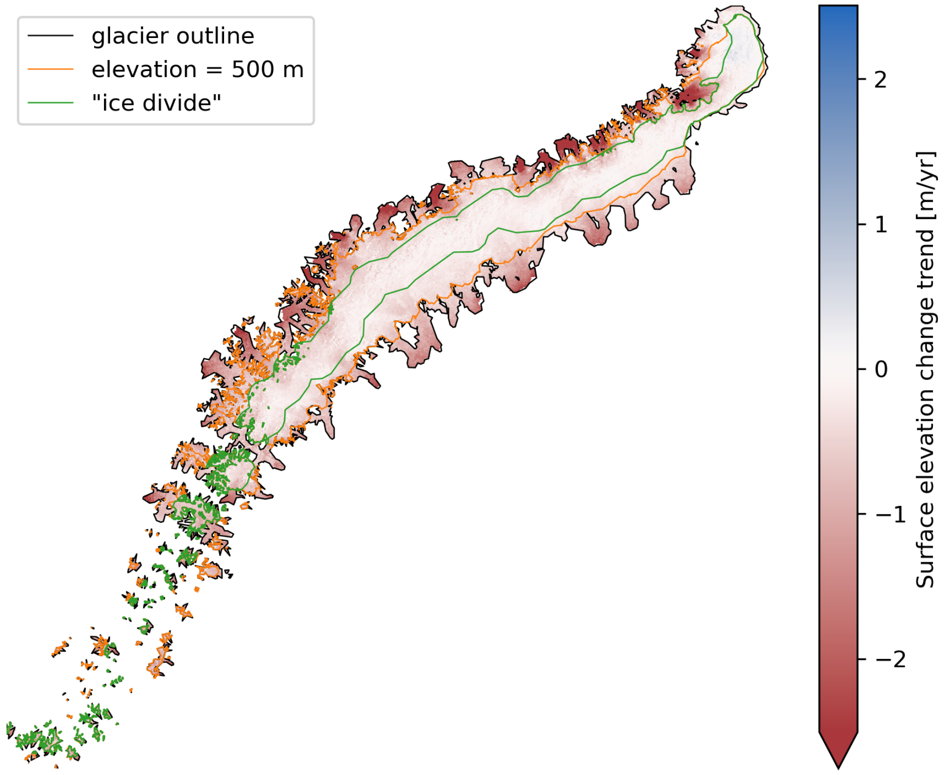


Fig. S3: CryoSat-2-observed average surface elevation change rates 2011–2022. The black, orange, and green outlines indicate the total glacierized area, the 500 m-elevation line, and the "ice divide" (in analogy Moholdt *et al.* [1]) as used in Table S2.

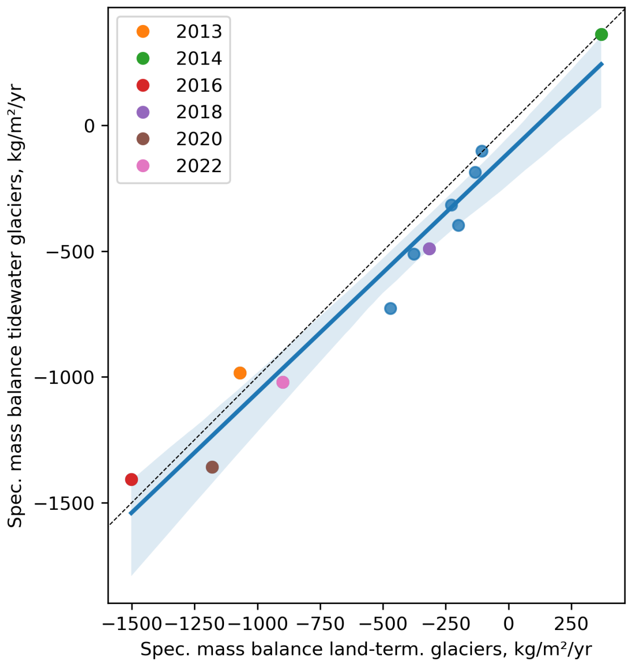


Fig. S4: Comparison of yearly area-specific mass changes of marine- and land-terminating glaciers. Those years that were mentioned in the main article can be identified using the legend; other years are colored in blue.

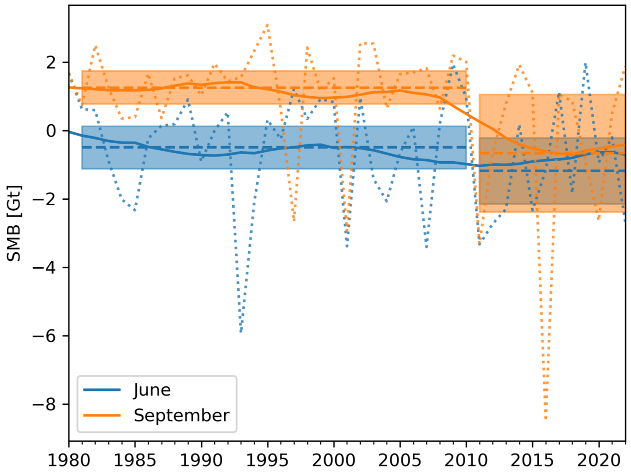


Fig. S5: June and September MAR SMB including averages for the periods 1981–2010 and 2011–2022. The dotted line shows the modeled values for each year and the solid line the low-pass filtered version thereof. The dashed lines and shaded areas show the averages and their $2\sigma$-confidence intervals for the periods 1981 to 2010 and 2011 to 2022.

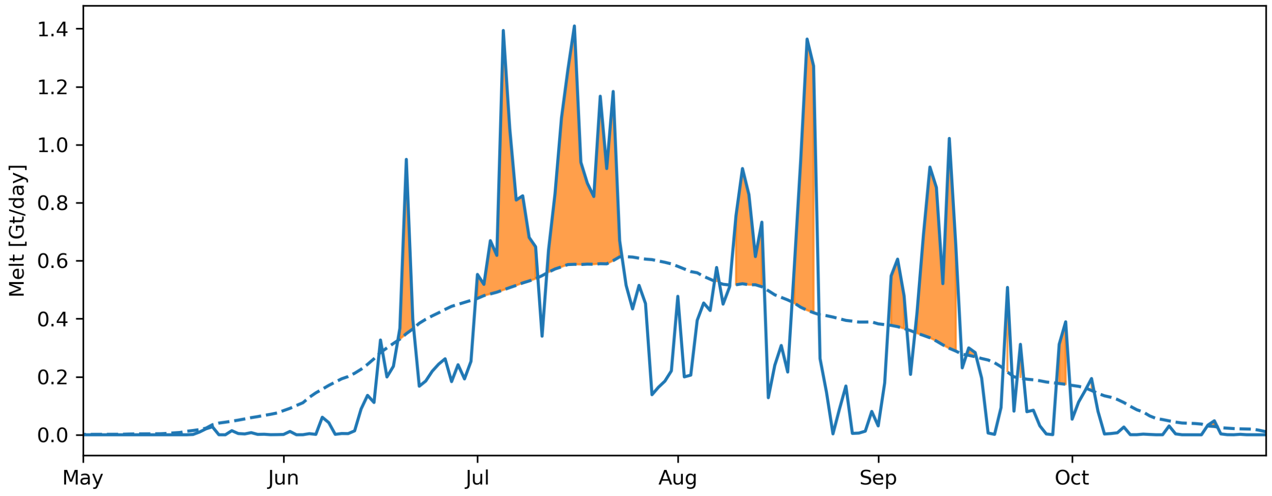


Fig. S6: Daily MAR melt in 2016. Dashed line represents the low-pass filtered version (gaussian window, $\sigma = 31$ days). Orange areas highlight the daily melt above its low-pass filtered version.

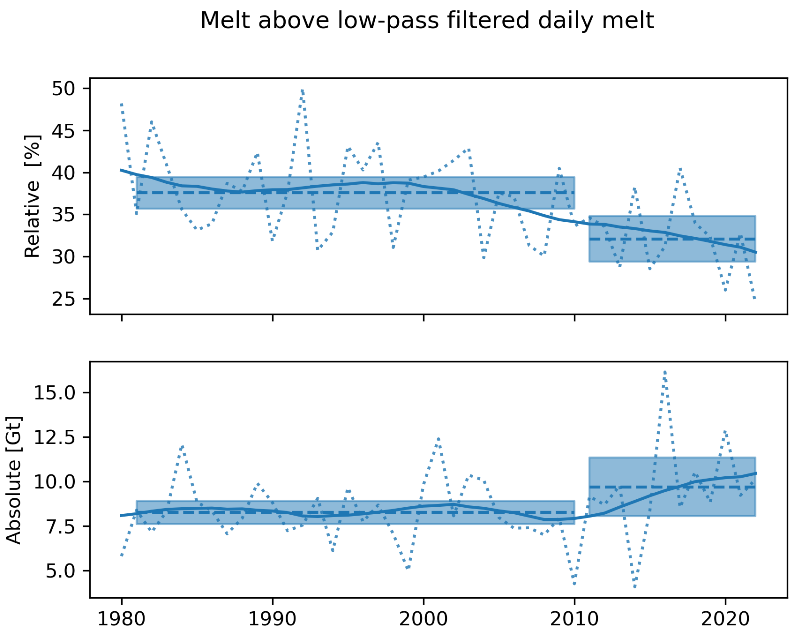


Fig. S7: Melt above its low-pass filtered version (cmp. orange highlighted areas in Fig. S6). Dotted lines show raw data, solid lines show a low-pass filtered version, dashed lines indicate the averages from 1981 to 2010 and 2011 to 2022, and the shaded areas indicate the $2\sigma$-uncertainty intervals of the averages.

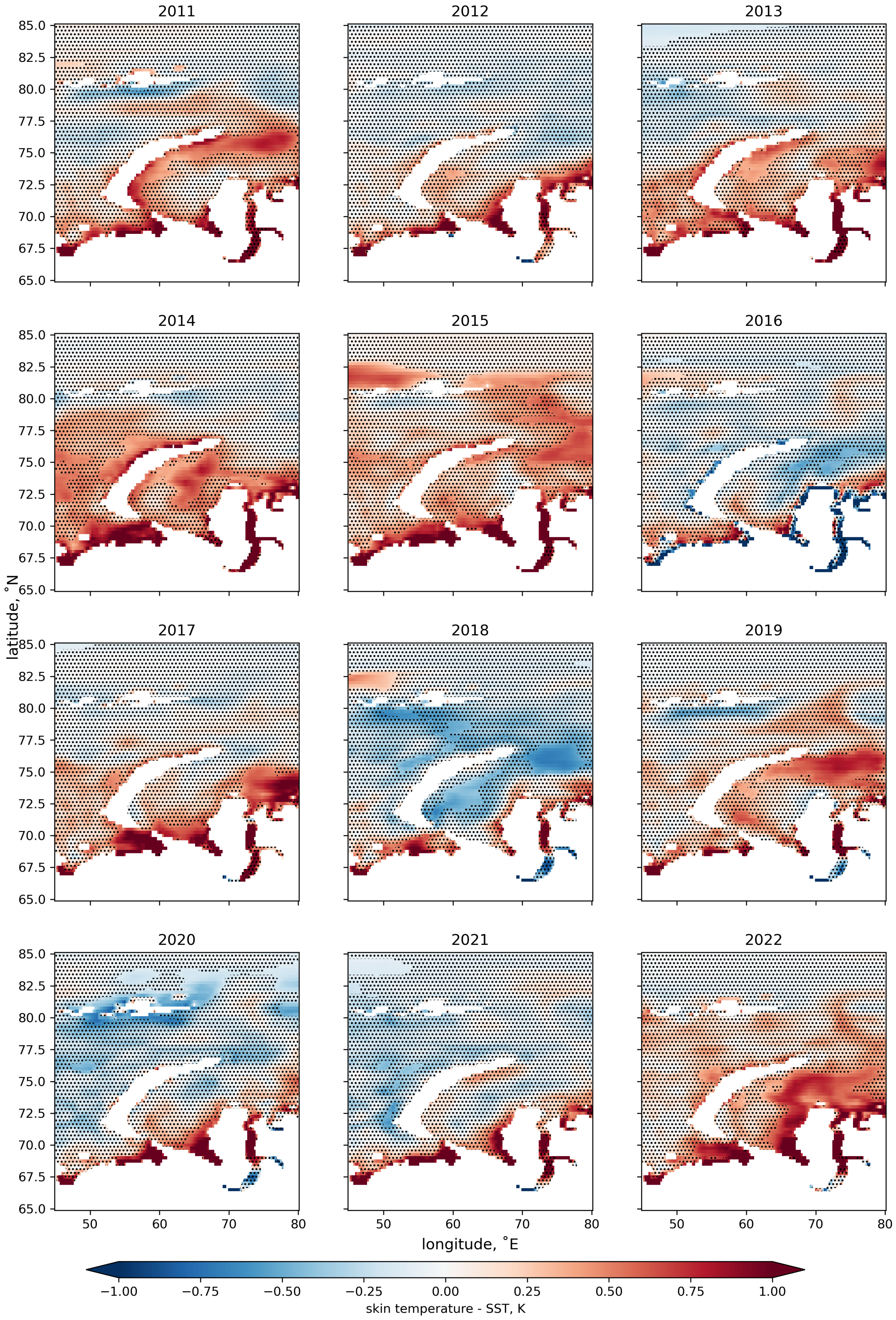


Fig. S8: Comparison of the melt-season (Jun–Sep) averages of the sea surface skin temperature with the 2 m-air-temperature as difference of their anomalies (see Methods). Stippled areas are not significantly different from their 1981 to 2010 average.

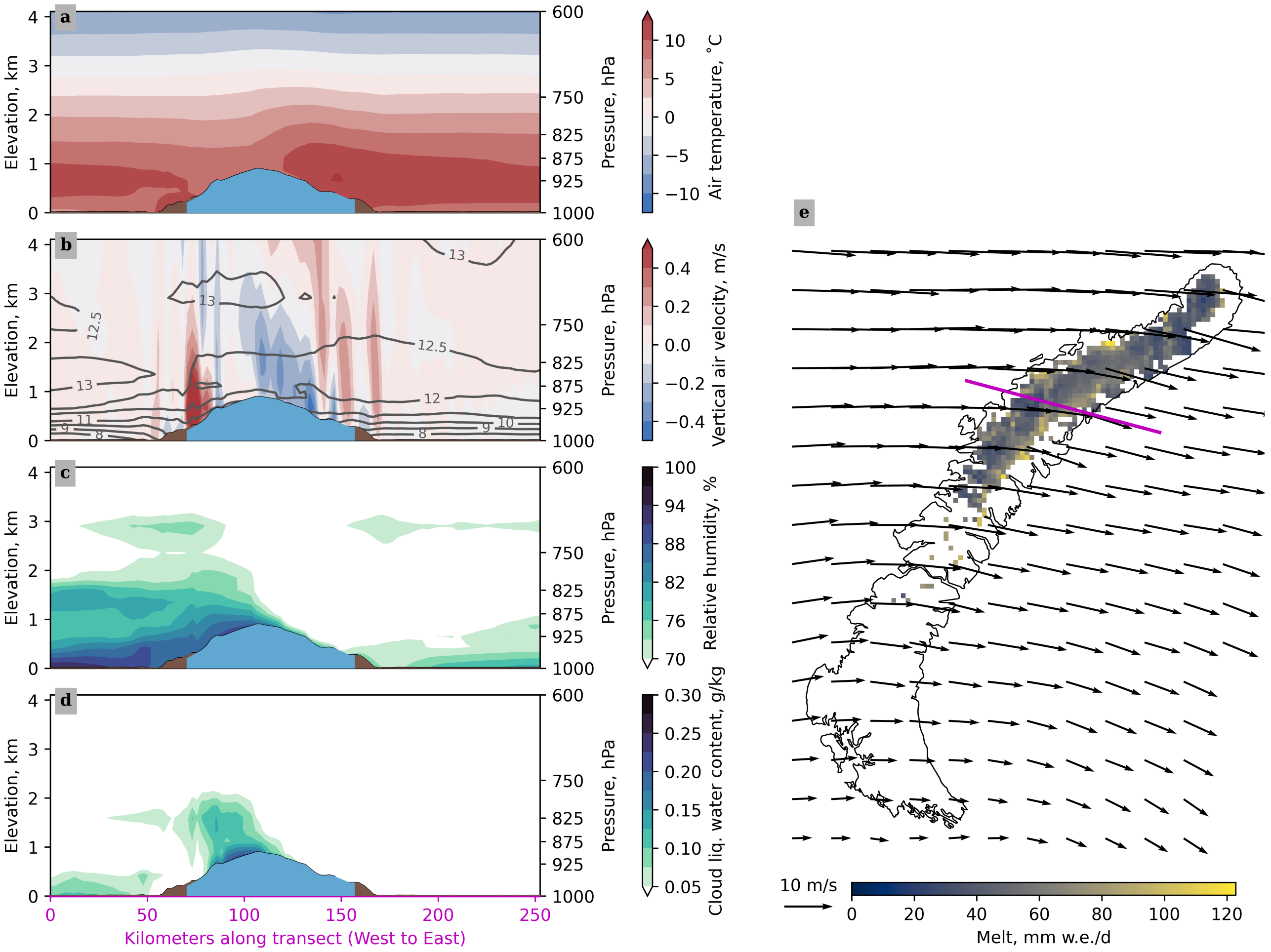


Fig. S9: In analogy to main article Fig. 4, this figure shows the average conditions at 12 UTC for 12 days with westerly winds and MAR melt exceeding 1 Gt (see Table S3). The panels show the CARRA (a) air temperature, (b) vertical air velocity, (c) relative humidity, and (d) cloud liquid water content along the transect highlighted in panel (e) from west to east. In panels (a-d), the brown and blue colored shapes indicate land and glacierized areas, respectively. The contourlines in panel (b) show isotherms of the potential irreversible moist-adiabatic temperature in °C. Panel (e) shows the average CARRA wind speeds at 850 hPa, the average MAR melt, and the coastline of Novaya Zemlya.

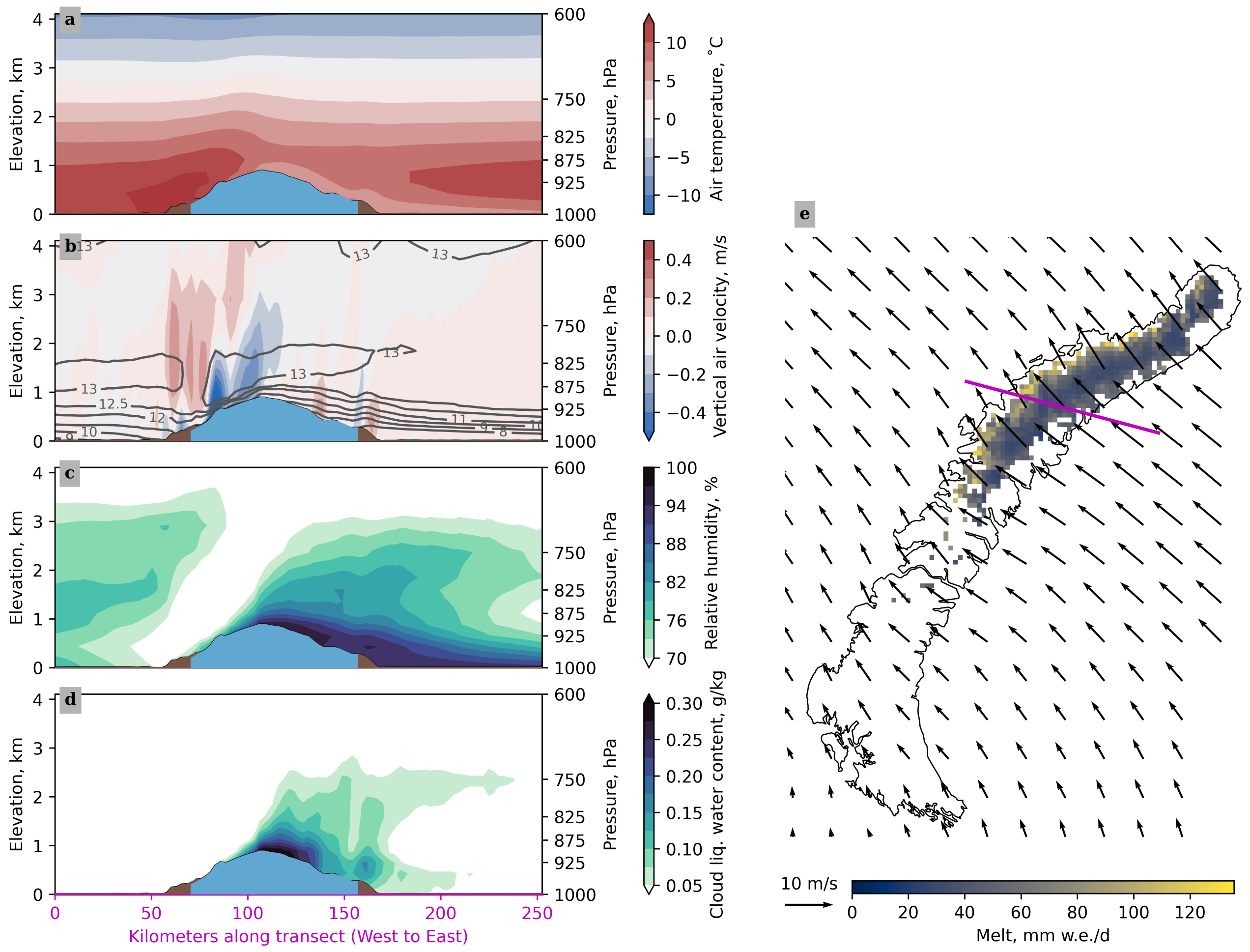


Fig. S10: Same as Fig. S9, but for 32 days with easterly winds.

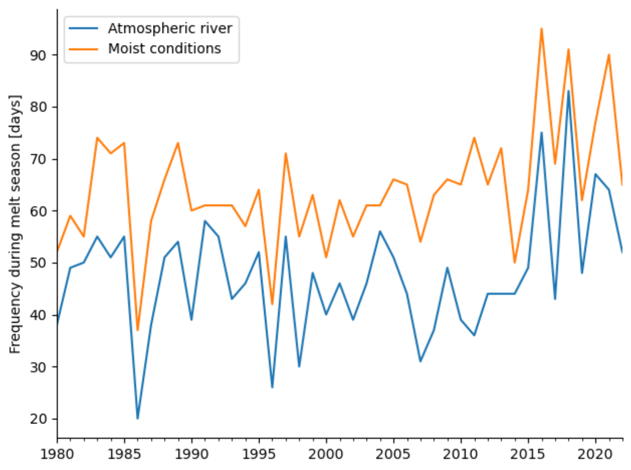


Fig. S11: Comparison of number of days per year with an AR[2] closer than 50 km or moist conditions (see main article).

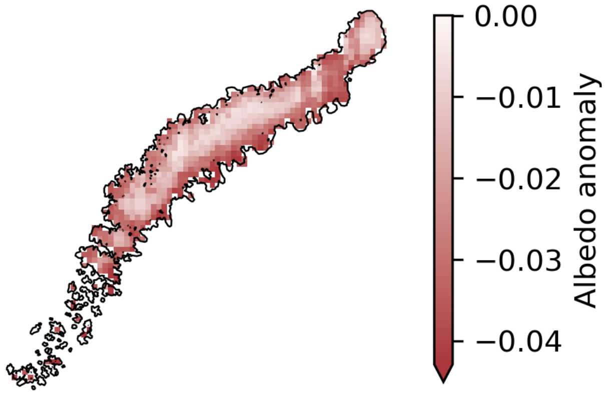


Fig. S12: 2011–2022 average melt-season MAR albedo anomaly.

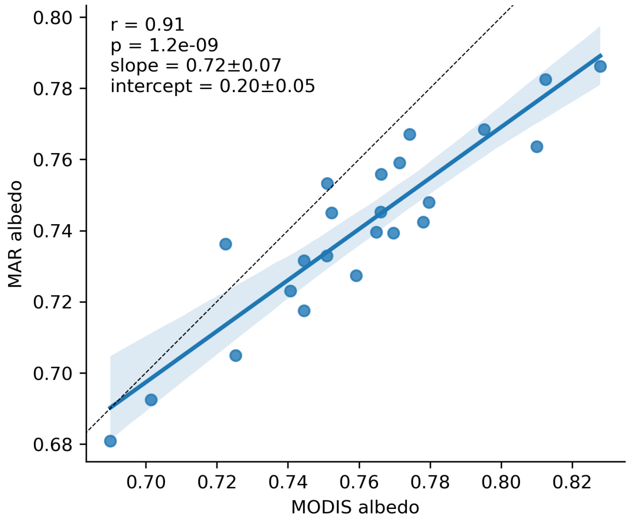


Fig. S13: Comparison of yearly melt-season albedo average from MAR and MODIS. The thin black dashed line indicates the identity line.

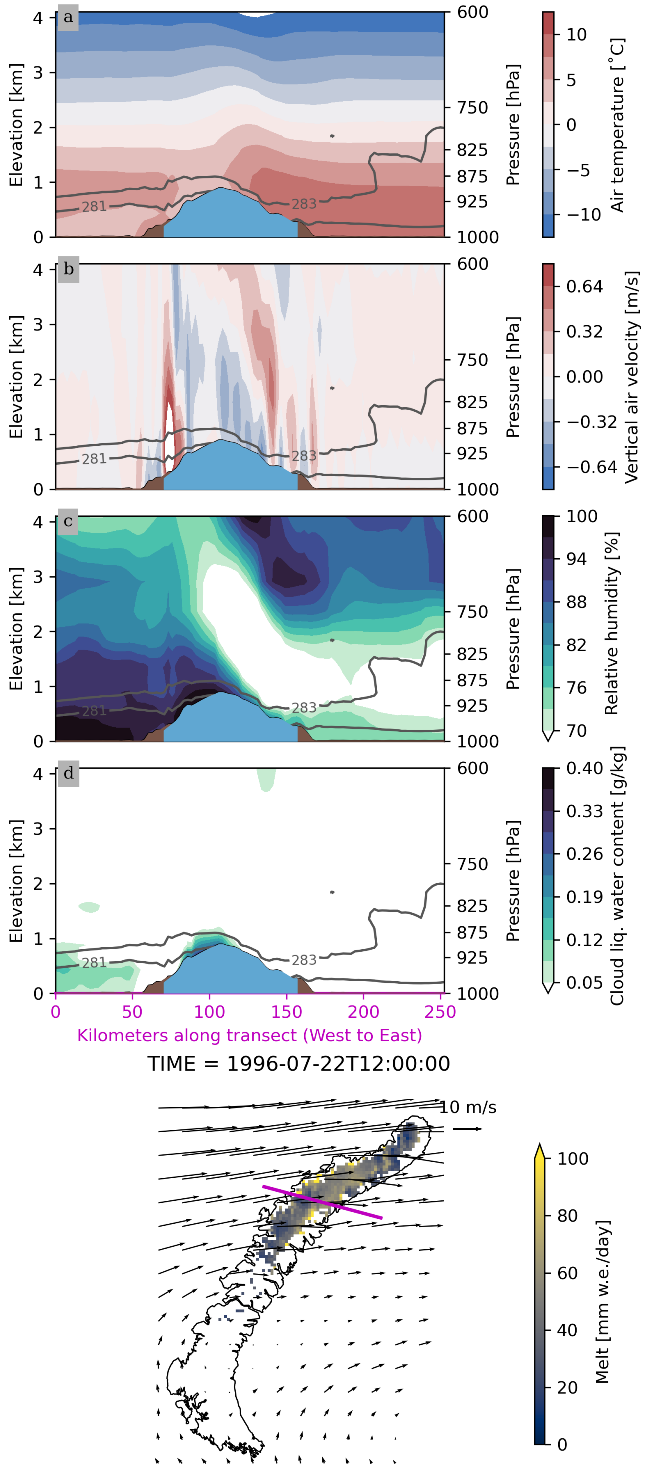


Fig. S14: The panels show average CARRA (a) air temperature, (b) vertical air velocity, (c) relative humidity, and (d) cloud liquid water content on 22 July, 1996, along the transect highlighted in the unlabeled bottom panel from west to east. In panels (a-d), the brown and blue colored shapes indicate land and glacierized areas, respectively, and the contourlines show isotherms of the potential irreversible moist-adiabatic temperature in K. The bottom panel shows the average CARRA wind speeds at 850 hPa, the average MAR melt, and the coastline of Novaya Zemlya.

## References

1. Moholdt, G., Wouters, B. & Gardner, A. S. Recent mass changes of glaciers in the Russian High Arctic. *Geophysical research letters* **39** (2012).
2. Mattingly, K. S. *et al.* Increasing extreme melt in northeast Greenland linked to foehn winds and atmospheric rivers. *Nature communications* **14,** 1743 (2023).
